# Levetiracetam (levebel) Versus Carbamazepine Monotherapy for Focal Epilepsy in Children: A randomized clinical trial

**Published:** 2020

**Authors:** Javad AKHONDIAN, Farah ASHRAFZADEH, Hossein ESLAMIYEH

**Affiliations:** 1 Department Of Pediatric Neurology , Quaem Hospital ,Mashad University of Medical Sciences ,Mashad ,Iran; 2 Department Of of Pediatric Neurology, Shahid Sadoughi University of Medical Science ,Yazd, Iran

**Keywords:** Childhood focal epilepsy, carbamazepine, levetiracetam, pediatric effectiveness

## Abstract

**Objective:**

This study aimed at comparing the effect of a newly approved drug leveitiracetam (LEV) versus carbamazepine (CBZ) in the treatment of childhood focal epilepsy.

**Methods & Materials:**

The study population included newly diagnosed children with focal epilepsy (1-16 years old) referring to the Pediatric Neurology Ward of Quaem Hospital, Mashhad, Iran from May 2013 to March 2014. The subjects were randomly treated with LEV or CBZ. Patients were followed for seizure control and drug side effects throughout six months. We assessed liver function and complete blood count for all patients through one month and they were asked about significant side effects, such as drowsiness، restlessness, and skin reaction. Eventually, they were assigned in two groups (n=25) receiving LEV and CBZ.

**Results:**

In our study, two cases in the LEV group were excluded because of severe agitation. Relapsing seizures were observed in 3 (13%) and 10 (40%) patients in LEV and CBZ groups, respectively. The seizure was not repeated in 15 cases (60%) in the CBZ group and 20 cases (87%) in the LEV group. The results of the Chi-squared test showed significant differences in the responses to treatment between the groups (*P*=0.03). Agitation was the most prevalent complication in the LEV group, whereas drowsiness was more common in the CBZ group. Fortunately, liver enzyme dysfunction and blood cell disturbances were not observed in the subjects.

**Conclusion:**

According to the findings, there were significant differences in controlling seizures between two groups that indicated the effectiveness of LEV (87%) in the suppression of focal seizure.

## Introduction

Epilepsy is a demanding neurological condition that affects many people worldwide. Selecting an appropriate antiepileptic drug (AED) is still challenging, because the selected drug should be effective, safe, and tolerable. Older generation of AEDs, such as phenobarbital and phenytoin are not widely accepted as a primary monotherapy and also long-term therapy for focal seizures, because of their side effects (1).

This problem is more common in pediatrics, particularly those over the age of one year. Only topiramate and oxcarbazepine are approved as monotherapy despite their side effects, such as leukopenia, aplastic anemia and drug-induced hepatitis. Because these drugs have the potential for drug interactions, reducing the serum level of other AEDs, and producing drug - drug interaction, it is important to consider the safety and efficacy of an AED separately for monotherapy and adjunctive therapy(2).

The newer generation of AEDs have often more favorable side effects, including lesser somnolence and blood dyscrasia than the traditional AEDs. However, no comparative study has demonstrated the improved efficacy over carbamazepine (CBZ), phenytoin or valproic acid (3).

Levetiracetam (LEV), the *S*-enantiomer of alpha-ethyl-2-oxo-1-pyrrolidine acetamide, is a novel AED that has been approved for use as an add-on therapy for partial-onset seizures in children older than one year. In addition, LEV may provide effective seizure control when used as monotherapy (4). No serious toxicity has been reported for LEV (5). LEV does not affect the liver enzymes, like CYP450. Hence, there is no report on its major interaction with other AEDs (2).

Little evidence is available for LEV monotherapy in children younger than 16 years (6(. Although several other studies have demonstrated successful conversion to monotherapy in a small number of children, the response rate with various durations of treatment in children with refractory epilepsy was as high as 66% (7,8).

To date, there are limited comparative findings regarding older and newer generations of AEDs (9) and there is no prospective study for this comparison.

This study aimed at comparing the effects of LEV and CBZ as monotherapy in children with focal seizures.

## Materials & Methods

This Single-blind, randomized, prospective study (data recorder was blind to the drug administration) was conducted among 50 newly diagnosed children having focal epilepsy and referring to the Quaem Hospital Pediatric Neurology Ward, Mashhad, Iran from May 2013 to March 2014. The age range of patients was 1-16 years. 

Research protocol was approved by the Ethics Committee of the Mashad University of Medical Sciences (t-3181) and the written informed consent was obtained from the parents of the subjects.

***Patients. ***The age range of 1-16 years, newly diagnosed focal epilepsy, no history of refractory seizures, the lack of other systemic underlying disorders, especially renal, hepatic, or brain diseases, such as cerebral palsy and no history of previous AED use were the inclusion criteria. Those with pseudo- seizures, drug reaction and major side effects, such as Stevens-Johnson syndrome, drug-induced hepatitis, psychosis, renal disorders, severe agitation or any other minor problems, the lack of parents’ willingness to participate in the study and clinical or electroencephalographic findings suggestive of idiopathic generalized epilepsy were the exclusion criteria. 

***Study design. ***The study participants were randomly assigned to the two treatment groups receiving either LEV or CBZ. LEV (Levebel) was initiated at an initial dose of 10mg/kg/d and increased by 10mg/kg weekly until it reached the usual dose of 30mg/kg/d and continued. In the other group, CBZ (Loqman) was initiated at an initial dose of 5mg/kg/d and increased by 5 mg/kg weekly until it reached the usual dose of 15mg/kg/d and then continued.

At first, all participants were subjected to electroencephalography (EEG). To evaluate hepatic and hematologic side effects, complete blood count (CBC), alkaline phosphatase (ALKP) and aminotransferases (AST and ALT) tests were done one month later. Participants were assessed for side effects, such as somnolence, agitation, urticaria or skin itching. The patients then were divided into two 25-member groups, one group received LEV and the other one received CBZ. These groups were then sub-divided into two groups: responsive and non-responsive to therapy. Patients who completed the trial were considered to receive the allocated treatment until data analysis.

***Statistics***

Due to the lack of relevant study, this study was done as a pilot research. The subjects were initially dichotomized into two groups: those who treated with LEV and those with CBZ. The Student *t-*test and Chi-square test were used to compare continuous parametric and nonparametric data, respectively. The Fisher's exact test was used for categorical variables. The seizure-free period was calculated for each subject. The occurrence of adverse events was compared between the two treatment groups using the dosage at the onset of the adverse events and the interval between the initiation of the AED administration and the occurrence of the adverse events. 

## Results

The initial evaluation sowed 25 patients with seizures who were younger than 16 years treated with LEV and 25 cases treated with CBZ who met inclusion criteria. The demographic characteristics of the two treatment groups were comparable and all patients were followed for 6 months after the initiation of monotherapy (Table 1). Two participants receiving LEV were excluded from the study because they developed severe agitation. The final analysis was done on 48 participants. No other case was excluded to follow-up or discontinued taking the medication. There was no need to add adjunct AEDs during the follow-up period.

The mean age of the participants was 7.32±3 years in the CBZ group and 7.89±2.5 years in the LEV group. Based on the Independent sample *t*-test, there was no significant difference in terms of age between the groups (*P *value: 0.516). Twenty-three participants (47.9%) had normal EEG (12 participants in the CBZ group and 11 in the LEV group), whereas 25 participants (52.1%) had abnormal EEG (13 patients in the CBZ group and 12 patients in the LEV group). Chi-square test revealed no significant difference in the frequency of participants with normal and abnormal EEG between the two groups (*P* value: 0.990(. In the CBZ group, 10 participants (40%) (or 20.8% of the total participants) did not respond to the therapy and had one or more seizures during the follow-up period. In the LEV group, only three (13%) participants (6.3% of the total participants) did not respond to the therapy. Fifteen (60%) participants receiving CBZ and 20 participants (87%) receiving LEV responded to the therapy.

There was no significant difference between the participants who were free of seizure attacks during a six-month follow-up and those who had seizure attacks. Regardless of the seizure type, Chi-squared test revealed a statistically significant difference in the response to the therapy between the CBZ and LEV groups (*P* value: 0.035). The participants on LEV monotherapy had a significantly higher response rate. Moreover, in the LEV group, there was no significant difference between the participants who were free of seizure attacks through a six-month follow-up and those who were not.

In the CBZ monotherapy group, five participants (20%) had a complex partial seizure and 20 subjects (80%) had secondary generalized seizures. Moreover, in the LEV monotherapy group, 5 participants (21.7%) had complex partial seizures and 18 subjects (78.3%) had secondary generalized seizures during the follow-up period. Chi-square test revealed no statistically significant difference in the frequency and type of seizure between the two groups (*P* value: 0.882).

Totally, 16 subjects (32%) out of the 50 participants [9 (36%) on CBZ and 7 (28%) on the LEV] experienced at least one adverse event and none of the adverse events were life-threatening. Of the total participants, 68% subjects (34/50) did not show any adverse events [16 cases (64%) in the CBZ group and 18 cases (72%) in the LEV group]. in addition, the Chi-squared test revealed no statistically significant difference in the occurrence of complications between the two groups (*P* value 0.853).

Six (12.5%) participants in the CBZ group reported somnolence and impaired consciousness; however, no somnolence sign was reported in the LEV group. The Chi-squared test results showed a statistically significant difference between the two groups (*P* value: 0.012).

There were no reported agitation signs in the CBZ group, whereas 7 cases (30.4% of the total) in the LEV group reported agitation signs. Considering those who were excluded, 7 out of the 25 participants (36%) in the LEV group had agitation signs. The Chi-squared test results revealed a statistically significant difference in agitation signs between the two treatment groups (*P* value: 0.003).

On the other hand, dermatologic and hepatic complications were reported only in the CBZ group. However, there were no statistically significant differences in the occurrence of these side effects between the two groups.

Only one participant in the CBZ group developed a hepatic complication. Liver enzymes were three times more than the upper limit. The liver function test repeated one week later revealed that the level of the enzyme had returned back to the normal range, thus, the treatment was continued. No hepatic complication and skin reactions were seen or reported in the LEV group. Only two participants in the CBZ group developed skin complications (itching and redness) who were treated with anti-histaminic medications. There was no hematologic complication among the participants in both groups. Furthermore, data analysis showed no statistically significant in all complications between the two groups.

## Discussion

It is difficult to design trials on the AED monotherapy to demonstrate its use in clinical practices in children with focal seizure (10). LEV is a novel medication that has been approved by the Food and Drugs Administration (FDA) for adjunctive therapy in children older than one month with focal seizure (11).

Few studies have specifically examined LEV monotherapy in children with newly diagnosed focal epilepsy, whereas many studies have suggested the efficacy of LEV as add-on therapy in adults and children (4,6).

This was the first randomized clinical trial that compared the efficacy of LEV with CBZ monotherapy in children. In this study, we investigated the efficacy of LEV compared with CBZ regarding the newly diagnosed focal epilepsy. The main goal of the study was the elimination of the seizure for at least 6 months after treatment initiation and continuing the therapy with LEV or CBZ, which can indicate the efficacy of the medication. The standard formulation of the CBZ and LEV were used with a fixed starting dose, slow titration, and the possibility for patients to remain on the modest effective dose.

The primary outcome of this study was seizure remission for at least 6 months after the initiation of therapy with LEV or CBZ. More than 70% of the participants responded positively to these monotherapies and had no seizure through a six-month follow-up, which is consistent with the results of Perry et al. (2008) study (11). Our results showed more effectiveness in participants receiving LEV with 87% reduction in seizure frequency than to those receiving CBZ (*P* value: 0.035). Seizure freedom may be a more reliable measure for a prospective review, as it is often more clearly documented in the chart and is less susceptible to the recall bias resulting from the reliance on seizure-frequency counts provided by the parents. In the present study, all participants were subjected to monotherapy for at least six months. The majority of participants (87%) receiving LEV, (20/23 participants) experienced seizure freedom through a six-month follow-up. This proportion was significantly higher than the participants in the CBZ group (60%) who were seizure-free during the follow-up period. A previous study on 18 children treated with LEV did not report information about the conversion, treatment initiation, duration of treatment, and clinical response (12).

Another relevant study reported that 73% of the participants receiving LEV and 65% treated with CBZ were free of seizures. However, the proportion of those treated with CBZ who were free of seizures was slightly higher than our findings (11). Another study reported no statistically significant difference in seizures freedom between participants treated with LEV and CBZ (3). In contrast, our findings revealed a statistically significant difference in seizure remission between the participants in the LEV and CBZ groups (*P* value: 0.035). Other studies also reported a higher proportion of seizure-free response among participants with partial seizure who received LEV monotherapy (8,9). Furthermore, 57% of the participants (children younger than 4 years) who were treated with LEV or CBZ monotherapy for focal seizures have been reported to be free of seizures during the first six months of follow-up (13).

We prescribed LEV at an initial dose of 10 mg/kg/d that was increased to 30 mg/kg/d and continued. Another study also suggested a lower dose of LEV (≤30 mg/kg/d). Ben-Menachem et al. reported that changing the LEV therapy from adjunctive therapy to monotherapy at a dose of 1500mg twice a day was effective in reducing or ceasing the seizures attack (15).

Studies on LEV monotherapy in adults and children have reported the effectiveness at relatively lower doses of LEV (12,13).

These studies also suggested LEV as an effective medication for seizure remission in childhood focal epilepsy. Among the patients treated with LEV (12,13,15), there were no significant differences in seizure type. Both CBZ and LEV were well tolerated as an initial monotherapy (12,13).

Only 30% of the participants receiving LEV and 28% treated with CBZ had side effects. The findings showed no statistically significant difference in the occurrence of side effects in the studied groups. This may be due to the small sample size in our study. Besides, only two participants in the LEV group discontinued the monotherapy because of severe agitation. There were no other side effects leading to the discontinuation of therapy among the treatment groups. Similarly, another study reported that agitation was the most common side effect that caused treatment cessation (15).

In our study, all other side effects, such as hepatic dermatologic diseases and somnolence were only seen in those receiving CBZ. Somnolence was a more frequent complication in patients receiving CBZ that occurred at relatively normal daily doses. However, serum CBZ levels were not routinely available. As a result, we could not determine the effect of dosages on plasma concentrations in participants reporting these complications. Similarly, somnolence was the more frequent side effect among patients treated with CBZ (11). However, the side-effect profile of a particular formulation, such as the controlled-release formulation of CBZ, which may be better tolerated, was not determined in this study. A statistically significant difference in agitation side effect was also found between the two groups (*P* value: 0.012), which is consistent with the findings of other studies (16-20).

It is recommended to conduct more studies using a larger sample size to compare the presence of intolerable side effects more accurately between the two groups receiving the specified monotherapies. It can provide an appropriate finding regarding the effectiveness of a specific therapy more specifically . Our study indicated a significant difference in the effectiveness of the LEV as the first-line medication compared with the CBZ. This implies the noninferiority to CBZ and a more favorable effectiveness of the LEV as a monotherapy for focal seizures. However, data analysis showed no statistically significant difference between the two groups.

To select a treatment for a patient with newly diagnosed epilepsy, the side effects and long-term safety should be considered. LEV can probably be a proper substitution for CBZ because it is associated with lower side effects, more safety, tolerability, and simpler pharmacokinetics that makes it a promising AED to be used as initial monotherapy in newly diagnosed epilepsy cases. The current study showed that children with newly diagnosed focal epilepsy treated with LEV had a better positive prognosis than those receiving CBZ. 

**Strengths and Limitations**

The use of seizures remission as an ultimate goal of treatment and as a marker of efficacy, the relative homogeneity between the groups, and using prospective design (randomized controlled trials) were the strengths of this study. However, this study was a pilot study using a small sample size. Hence, the direct comparison between the two groups with the antiepileptic monotherapies was limited. This study can provide strong findings for future trials on LEV monotherapy with a larger sample size in children younger than 16 years with focal epilepsy.

**Table 1:** Characteristics and adverse Event Profile of the patients receiving Levetiracetam and Carbamazepine

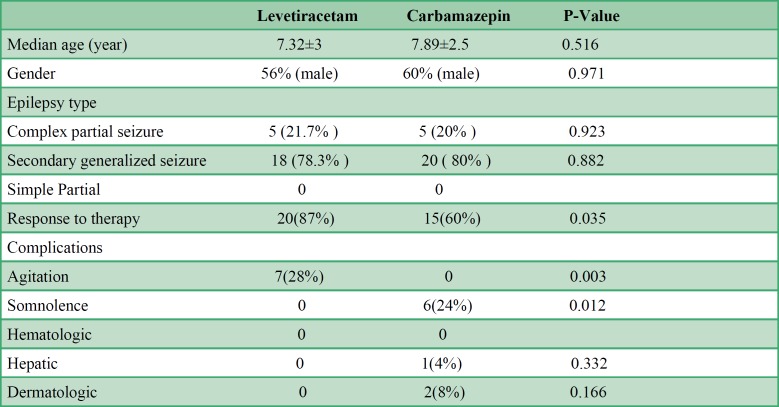

**In conclusion:**

To select a treatment for a patient with newly diagnosed epilepsy, the side effects and long-term safety should be considered. LEV can probably be a proper substitution for CBZ because it is associated with lower side effects, more safety, tolerability, and simpler pharmacokinetics that makes it a promising AED to be used as initial monotherapy in newly diagnosed epilepsy cases. The current study showed that children with newly diagnosed focal epilepsy treated with LEV had a better positive prognosis than those receiving CBZ. 

The use of seizures remission as an ultimate goal of treatment and as a marker of efficacy, the relative homogeneity between the groups, and using prospective design (randomized controlled trials) were the strengths of this study. However, this study was a pilot study using a small sample size. Hence, the direct comparison between the two groups with the antiepileptic monotherapies was limited. This study can provide strong findings for future trials on LEV monotherapy with a larger sample size in children younger than 16 years with focal epilepsy
